# Compassion Fade: Affect and Charity Are Greatest for a Single Child in Need

**DOI:** 10.1371/journal.pone.0100115

**Published:** 2014-06-18

**Authors:** Daniel Västfjäll, Paul Slovic, Marcus Mayorga, Ellen Peters

**Affiliations:** 1 Decision Research, Eugene, Oregon, United States of America; 2 Linköping University, Department of Behavioral Sciences and Learning, Linköping, Sweden; 3 University of Oregon, Department of Psychology, Eugene, Oregon, United States of America; 4 Ohio State University, Department of Psychology, Columbus, Ohio, United States of America; University of Vienna, Austria

## Abstract

Charitable giving in 2013 exceeded $300 billion, but why do we respond to some life-saving causes while ignoring others? In our first two studies, we demonstrated that valuation of lives is associated with affective feelings (self-reported and psychophysiological) and that a decline in compassion may begin with the second endangered life. In Study 3, this fading of compassion was reversed by describing multiple lives in a more unitary fashion. Study 4 extended our findings to loss-frame scenarios. Our capacity to feel sympathy for people in need appears limited, and this form of compassion fatigue can lead to apathy and inaction, consistent with what is seen repeatedly in response to many large-scale human and environmental catastrophes.

## Introduction

In a rational world, as threats to life increase in scale, potential efforts to prevent harm should increase proportionally [1]. Decisions at organizational and political levels regarding the allocation of resources for humanitarian aid are characterized by how the need of others is perceived [2]. Public policy decisions reflect public opinion on specific issues, and individual responses towards humanitarian crises are likely informing and guiding these decisions [3], [4]. The main tenet of the present research is that compassion and therefore societal concern often decrease rather than increase in the face of greater threats. The primary aim of the present article is to understand the psychological underpinnings of this perverse phenomenon. More specifically, we propose and test the hypothesis that the needs of others induce affective feelings, and that donors often experience the strongest feelings for a single identified person in need. As the number of needy persons increases, affective feelings and action may begin to diminish. Such “compassion fade” has implications for traditional theoretical models of valuation and, more broadly, for the welfare of society.

### Traditional Models for Valuing Human Lives

**Figure 1:**
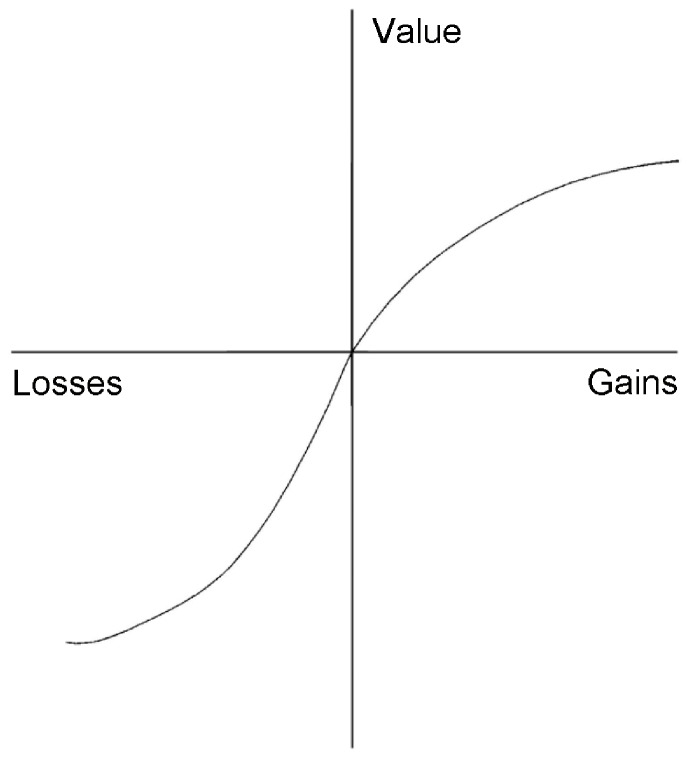
Prospect theory’s value function for gains and losses.

How do we value the potential or actual loss of lives? Egalitarian, normative models of life saving declare that every human life should be valued equally, yet psychological descriptive theories suggest that this is not the case [4]. For instance, prospect theory [5], arguably the most important descriptive theoretical framework in the field of decision making, proposes that the carriers of value are positive or negative changes from a reference point. The valuation function is nonlinear, reflecting diminishing sensitivity to magnitude characteristic of many psychophysical relationships. In the positive domain, for example, a gain of two tends to be valued as less than twice that of a gain of one (Figure 1). Referring to Figure 1, Kahneman [6] observed that “If prospect theory had a flag, this image would be drawn on it” (p. 282). Applications of the theory have focused on gains and losses of money and human lives.

Consistent with the psychophysical function in prospect theory, Fetherstonhaugh, Slovic, Johnson, and Friedrich [7] found that the value of a life diminished against the backdrop of a larger tragedy. For example, imagine that one person is at risk of dying and that you could save her life by a donation to a trusted humanitarian aid organization. Now imagine that 87 other persons are at risk of dying, and that you could help save this same person (now the 88th) by a similar donation to her. Would you give the same amount in both scenarios? Even though in both cases the recipient is the same person, donations likely will be greater in the first scenario than in the second [1], [8].

We propose the need to modify the value function in contexts pertaining to the value of saving human lives as the number of lives at stake increases. Although we do find evidence supporting the monotonically increasing function with diminishing sensitivity depicted in Figure 1, it may be that, in some contexts, a decrease in valuation better describes how we react to greater needs.

### Compassion Fade: A New Descriptive Model for Valuing Lives

Affective feelings such as empathy, sympathy, sadness, and compassion are often seen as essential for motivating helping [9], [10]. Emerging evidence from neuroscience research also supports the notion that affective feelings are integral to charitable giving [11]–[13]. Slovic [1] suggests a model in which mental images and attention are the precursors for affective feelings toward others in distress. Consistent with this model, Dickert and Slovic [14] demonstrated that sympathy towards a child in need was greater when attention was focused directly on the child than when judgments were made from memory. Distractor children in the visual field also reduced sympathy toward the target child.

We propose that decisions about saving lives depend heavily on affect. Affect, as defined here, is a feeling (not necessarily conscious) that something is good or bad. Affective responses occur rapidly and automatically. We, and others, have earlier suggested that affect has several functions [15]. One of them is to motivate behavior [16], [17], including decision making [18], [19]. Another is to add meaning to information [20]. Without affect, information lacks meaning and will not be used in judgment and decision making [21], [22]. Affect plays a central role in *dual-process theories* of thinking, which distinguish between experiential modes of thought and deliberative modes (sometimes labeled System 1/fast thinking and System 2//slow thinking, respectively; [6; for a discussion about different dual processes models in decision making see 23–25]). One of the characteristics of experiential thinking is its affective basis. Although deliberation is certainly important in many decision-making circumstances, reliance on affect and emotion as sources of information tends to be a quicker, easier, and more efficient way to navigate in a complex, uncertain and sometimes dangerous world [6].

In his Nobel Prize Address, Kahneman notes that the operating characteristics of System 1 are similar to those of human perceptual processes [26]. He points out that one of the functions of System 2 is to monitor the quality of the intuitive impressions formed by System 1. Kahneman and Frederick [27] suggest that this monitoring is typically rather lax and allows many intuitive judgments to be expressed in behavior, including some that are erroneous.

Importantly, System 1 appears to be limited in its capacity to deal with quantities. System 1 tends to be an on-off system driven, to a large extent, by images [2]. It is relatively insensitive to scope [28], [29]. For instance, Hsee and Rottenstreich [29] found that donations to help one versus four pandas were not significantly different when photos of the animals were shown to participants. Given that we assign affect a primary role in motivating actions, this dissociation between affect and abstract numbers is a problem for how we value the saving of human lives.

Initial evidence for this comes from research showing that compassion shown towards victims decreases as the number of individuals in need of aid increases [30], identifiability of the victims decreases [31], and the proportion of victims helped declines [7]. Such fading of compassion has the potential to significantly hamper individual-level and collective (e.g., political) responses to pressing large-scale crises, such as genocide or mass starvation [5] or severe environmental degradation [32].

### Singularity Effects in Charitable Giving

In this article, we examine how affective feelings driven by attention may underlie findings that, when it comes to eliciting compassion, a single individual with a face and a name typically evokes a stronger response than a group. Numerous studies have demonstrated the *identifiable victim effect,* which is also quite evident outside the laboratory. People are much more willing to aid one identified individual than to help numerous unidentified or statistical victims [33]–[35]. Why is this the case? Research by Hamilton and Sherman [36] and Thompson, Hamilton, and Rust [37] demonstrates that a single individual, unlike a group, is viewed as a psychologically coherent unit. This leads to more extensive processing of information and stronger impressions about individuals than about groups. Consistent with this, Kogut and Ritov [30] found that people tend to report feeling more distress and compassion when considering a single identified victim than when considering a group of victims, even if identified (e.g., a singularity effect).

Specifically, Kogut and Ritov asked participants to contribute to a costly life-saving treatment needed by a sick child or a group of eight sick children. The target amount needed to save the child (children) was the same in both conditions. All contributions were actually donated to children in need of cancer treatment. In addition, participants rated their feelings of distress (feeling worried, upset, and sad) towards the sick child (children). The sum of contributions to the single individuals far exceeded contributions to the group even though the group was comprised of some of these same individuals. Ratings of distress were also higher in the individual condition. The authors concluded that the greater donations to the single victim most likely stemmed from the stronger affective feelings evoked by such victims.

### Overview of the Present Studies

We use the term “compassion fade” to denote decreases in 1) behavior, and 2) affect when the number of needy individuals increase. “Compassion” is thus used to both capture the subjective and behavioral components of this phenomena. The term compassion is often used synonymously with terms such as empathy, sympathy and empathic concern in the literature [9], [38]. However, compassion and sympathy are typically other-oriented, whereas empathy is denoting sharing of affective experiences [39]. Consistent with some previous research, we treat compassion as a primarily positive valenced feeling state [40] that is outer-focused and motivates prosocial action [41] through activation of approach behaviors [42]. However, increases in the number of needy individuals, as well as personalized perceptions of single individuals, also increase personal distress and other negative self-focused emotions [38], [39], [43] which could activate withdrawal behaviors as well as approach behaviors [44]. In this study, we characterize compassion fade as loss of positive affect and diminished approach behaviors, consistent with recent theorizing [41] and empirical results from both self-reports and brain imaging [13]. We expect positive affect to be strongest for a single identified individual. Further, positive affective is expected to decrease as the number of needy individuals increase. This relative decrease in positive affect is expected to (de-)motivate behavior (or weaken approach behaviors). Thus, “compassion fade,” as used here, denotes decreases in positive affect that lead to decreases in donations, as the number of individuals in need increase.

In Studies 1–3, we examine the hypothesis that compassion fade may begin as early as with the second endangered life. We hypothesize that, as their numbers increase, less attention will be directed towards those in need and less feeling of attachment [45] and sympathy [30] will be experienced. As a consequence, we expect lower levels of positive affect towards the children (as indexed by self-reports [Studies 1a and 1b] and psychophysiological measures [Study 2]) and smaller donations as the number of children increases. In Study 3, we predict that positive affect and donations to a group of children would be increased by representing them as a single entity, rather than as multiple individuals. Finally, in Study 4, we examine compassion fade in the context of potential losses of life as opposed to life-saving opportunities.

#### Ethics statement

Experiments were conducted in accordance with the ethical standards laid down in the 1964 Declaration of Helsinki. Studies were approved by the local ethics committees where the data was collected (Västra Götalands regional ethics board, Studies 1–3, and IRB University of Oregon, Study 4). Participants were compensated for their participation and gave their informed consent prior to inclusion in the studies. In all studies, participants received information about the study prior to participating. After completing their task, participants were thoroughly debriefed.

### Studies 1a and 1b: Initial Demonstrations of Compassion Fade

In Studies 1a and 1b, we conducted initial tests of compassion fade by comparing ratings of self-reported affect and donations (hypothetical and real) for either a single needy child or two needy children (between-subjects design). Consistent with previous research on the singularity effect [30] the children were identified with a photograph, name, and age.

Based on the hypothesis that compassion fade may begin with the second child, we predicted that self-reported affect and donations would be higher for a single child than for two children. Statistical tests (planned contrasts using independent t-tests) comparing mean self-reported affect and donations between conditions were conducted to test this hypothesis. In addition, correlational and mediational analyses were conducted to examine the role of affect in motivating donation behaviors.

### Study 1a: Hypothetical Donations

#### Method

Two hundred and eight undergraduates in a Swedish university (106 males) with a mean age of 26.8 (*SD* 6.4) were presented with one of three scenarios. The two single-child conditions included a description and picture of either a seven-year-old girl, Rokia (*n* = 67) or a nine-year-old boy, Moussa (*n* = 69). Participants were instructed that:

Any money that you donate will go to Rokia [Moussa]. Rokia [Moussa] is desperately poor, and faces a threat of severe hunger or even starvation. Her [His] life will be changed for the better as a result of your financial gift. With your support, and the support of other caring sponsors, Save the Children will work with Rokia’s [Moussa’s] family and other members of the community to help feed her [him] and provide her [him] with education, as well as basic medical care and hygiene education.

In the two-children condition (*n* = 72), participants received a similar description but with pictures and stories of both Rokia and Moussa. Participants were instructed that their donations would go to Rokia *and* Moussa. Prior to the task, participants received information about the study and gave written consent to participate.

Three measures were used: 1) Willingness to donate. Participants could circle any number between 0 and 70 Swedish crowns (SEK) in 10-crown increments. 2) Affect. Feelings about donating were rated on a scale ranging from *slightly negative* (–1) to *positive* (5). 3) Probability that the donation would make a real difference (1 to 5 scale anchored by *not at all likely* to *very likely*).

#### Results and discussion

Contrasts comparing donation amounts showed that donations were significantly higher for the single conditions (*M* = 37.7 SEK) than for the two-children condition (*M* = 26.3 SEK), *t*(205) = 5.88, *p*<.001. Affect ratings were more positive in the single conditions (*M* = 3.7, vs *M* = 3.3; *t*(205) = 2.87, *p*<.01). The perceived probability of the donation making a real difference was higher in the single-child conditions (*M* = 4.4) than in the two-children conditions (*M* = 4.1); *t*(205) = 4.50, *p*<.001. A mediation analysis [46] showed that the effect of condition on donations was no longer significant (*b* = 0.09, *ns*) when affect (*b* = 0.47, *p*<.05) was entered as a mediator, (*F*(4, 201) = 9.98, *p*<0.01; Sobel *Z* = 3.0, *p*<.05). However, additional analyses showed that perceived probability did not mediate the effect of condition on donations (Sobel *Z* <1.0, *p*>.05).

### Study 1b: Real Donations

#### Participants and procedure

One hundred sixty-eight undergraduates in a Swedish university (66 males) with a mean age of 26.4 (*SD* 6.2) first completed an unrelated survey for which they received 70 SEK (seven 10 SEK coins), a blank envelope, a questionnaire, and a charity request letter. The experimenter instructed participants to first read the charity request letter carefully and place their donations (if any) in the envelope. Participants then were asked to complete the questionnaire and to return both the letter and questionnaire sealed in the envelope. The letter informed participants of the opportunity to donate any of their just-earned 70 SEK to the organization Save the Children. Following the donation decision, participants rated their feelings about donating to the child/children, ranging from *slightly negative* (–1) to *very positive* (+5).

As in Study 1b, participants were presented with one of three conditions. In the single-child conditions, they were given a description and picture of either a seven-year-old girl, Rokia (*n* = 47) or a nine-year-old boy, Moussa (*n* = 51). In the two-children condition (*n* = 70), participants received a similar description but with pictures and stories of both Rokia and Moussa presented simultaneously on the same page. Participants were instructed that their donations would go to Rokia *and* Moussa.

#### Results and discussion

**Figure 2:**
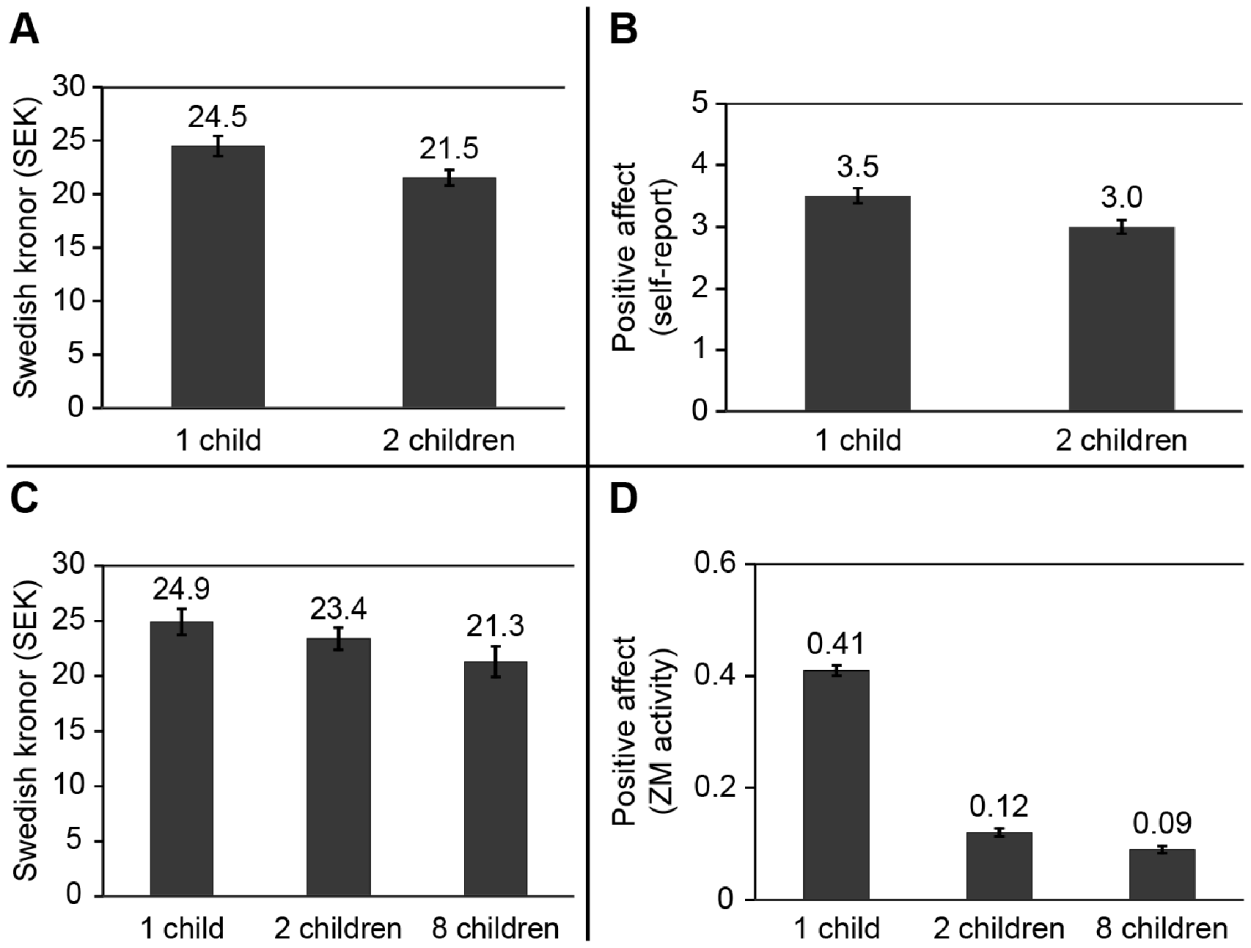
Real donations, self-report, and psychophysiological measures of affect for 1, 2, and 8 children. In Study 1, average donations (in SEK) decreased with increasing number of victims (A) and positive affect was stronger for the single victim (B). In Study 2, donations decreased with more victims (C) and positive affect (facial EMG measurement) decreased with an increasing number of victims (D). ZM activity for one child was significantly greater than for two children (*p*<0.05).

Planned contrasts showed that donations (*t*(166) = 1.67, *p*<.05) and ratings of affect (*t*(166) = 2.81, *p*<.01) were higher in the one-child (M = 24.5 SEK for donations, and M = 3.5 for affect ratings) conditions than in the two-children condition (M = 21.5 SEK for donations, and M = 3.0 for affect ratings; see Figure 2A and B). A mediation analysis [20] showed that the effect of condition on donations was no longer significant (*b* = 0.16, *ns*) when affect (*b* = 0.35, *p*<.01) was entered as a mediator, (*F*(4, 162) = 7.54, *p*<.001; Sobel *Z* = 2.1, *p*<.05). Studies 1a and 1b thus showed that self-reported affective feelings and donations to victims may start to decline as early as *N = *2. Across Studies 1a and 1b, self-reported positive affect and donations were higher in the single-child conditions compared to the two-children conditions. Further, the difference in positive affect, rather than perceived probability that the donation would help, mediated the effect of condition on donation amounts. Thus, Studies 1a and 1b are consistent with affect as a main driver of compassion fade.

### Study 2: Physiological Indicators of Compassion Fade

Study 2 aimed to replicate and extend the findings of Studies 1a and 1b. Participants were asked to donate to either one, two, or eight victims. Whereas affective feelings in Study 1 and in previous research on the identifiable victim and singularity effects have been measured using self-reports only, Study 2 incorporated physiological measures of positive affect. Specifically, activity in the facial Zygomaticus Major (ZM) was measured while participants viewed children in need and decided whether to donate or not. The ZM muscles are responsible for drawing back or tightening the cheek in smiling. Activity of the ZM muscles has been associated with self-reported pleasant emotions [47] and is expected with reactions to the children (feelings of compassion) or the anticipated warm glow of helping [48], [49]. We hypothesized that participants would experience stronger affect and donate more for the single child. To examine this hypothesis, we compared mean activity in ZM muscles (facial EMG) and donation amounts between the single-child, two-children, and eight-children conditions using within-subjects analysis of variance (ANOVAs). Further, correlational and mediational analyses were conducted to examine whether ZM muscle activity mediated the effect of condition on donations.

#### Participants and methods

One hundred seven undergraduates in a Swedish university (42 females) with a mean age of 29 years (*SD* = 4.4) completed the experiment.

A within-groups design was used whereby participants were asked to donate money to one, two, or eight identified children. The identified children were described with photographs, names, and background information. A randomized-block design was used such that participants saw eight separate presentations in each block (one, two, or eight children per presentation) presented sequentially (e.g., in the two-child block, participants were presented with eight different presentations each depicting two children). Within each block, the order of the stimuli was randomized. A Latin-square design was used to counterbalance the order between blocks. At the beginning of each block, participants were endowed with 70 SEK and were told they would be asked, at the end of the session, to donate money from this allocation based on one of their donation responses, selected randomly. The 70 SEK were given as seven 10 SEK coins and participants were instructed to place the amount they desired to donate for the selected trial in an envelope. Physiological measures of ZM activity were obtained for each stimulus shown in a block. In addition to ZM activity, measures of activity in the Corrugator Supercilii (CS) were also collected. CS activity has been linked to self-reported negative affect [47] and was included to obtain physiological markers of both positive affect (ZM activity) and negative affect (CS activity). However, based on Studies 1a and 1b showing that positive affect declined as the number of children in need increased, we primarily expected that ZM activity would differ between conditions. All physiological data were individually z-scored across subjects and, within a block, mean activity across the eight stimuli presented was used for final analysis.

Facial EMG recordings were first individually inspected for possible artifacts and were band-pass filtered from 10 to 400 Hz [47]. Change scores were calculated separately for each EMG signal by subtracting the average response for each one-second interval for the eight seconds following picture onset from the mean activity during the one second preceding picture onset (baseline). The average of the change scores from zero to eight seconds following picture onset was used in the subsequent analysis. There were order effects of presentation of the blocks such that donations and affect were higher for early donations (first or second presentation). However, no systematic interactions with condition were found.

#### Results and discussion

As in Study 1, donations decreased with an increasing number of victims (M_1 child_ = 24.9; M_2 children_ = 23.4, M_8 children_ = 21.3; linear trend analysis *F*(1,103) = 12.01, *p*<.001; see Figure 2C). ZM activity (positive affect) was highest for one victim and decreased linearly with increasing number of victims (M_1 child_ = .41; M_2 children_ = .12, M_8 children_ = .09, *F*(1,103) = 8.90, *p*<.001 (see Figure 2D). CS activity (negative affect) slightly increased at eight victims (M_1 child_ = .58; M_2 children_ = .47, M_8 children_ = .89) but this linear trend effect was not significant (*F* <1). To further examine the relationship between physiological indicators of affect and donations, we correlated ZM activity and donation amount within each condition. The correlation was strongest in the single-child condition (*r* = 0.44, *p*<.001), followed by the two-children condition (*r* = 0.26, *p*<.01), and the eight-children condition (*r* = 0.16, *ns*). A bootstrapped mediation analysis [34] tested whether donations were mediated by positive affect. It showed that the effect of condition on donations was no longer significant (*b* = 0.09, *ns*) when ZM activity (*b* = 0.48, *p*<.01) was entered as a mediator, Sobel *z* = 3.2, *p*<.001). An analysis with CS activity showed no mediation (Sobel *z* <1.0, *p*>.05).

This is the first study to show that a physiological marker of positive affect, EMG activity in ZM, can differentiate between the number of children in need. This finding replicates and extends previous research on the singularity effect [30] by showing that positive affect decreases with increasing number of children in need. Previous research suggested that affect plays a role in the singularity effect, but did not establish how differently valenced emotions influence behavior, and in which direction. Studies 1a, 1b, and 2 together provide evidence that a single child elicits greater positive affect and donations. The loss of positive emotion with an increasing number of children appears to be reducing donations.

In Study 3 we examined whether describing many children as a single group would reduce compassion fade and maintain donations for the many at the level of the single individual.

### Study 3: Reversing Compassion Fade Through Entitativity

Single, identified individuals receive more help than do a collective of many needy individuals [1], [2], [30]. But can the collective be individualized as a unitary entity–for example, a family, a village, a school, or even a country–in a way that induces the high level of support that goes to an individual person?

As noted above, Hamilton and Sherman’s work [36] on entitativity suggests that individuals and groups are processed differently. More specifically, perceivers attempt to form in-depth, organized, and coherent impressions of individual targets while groups are processed more superficially. Further, greater attention to the individual relative to the group may tend to result in stronger affective reactions to the individual.

A recent study by Smith, Faro, and Burson [50] manipulated entitativity in a charitable giving context by comparing how “unitization” of unrelated individuals could increase donations. They compared six unrelated children in need of aid to the same six described as siblings and found that the latter elicited far greater donations.

In Study 3 we aimed to replicate and extend this finding using a version of our compassion-fade paradigm. If a unitization manipulation would increase donations and positive affect for the type of small groups of needy children we present here, this would suggest that entitativity is central for compassion fade. Further, if unitization manipulations are effective, this may be a way to combat compassion fade.

In a between-subjects design, participants were asked to indicate how much they would be willing to donate either to a single victim, two unrelated victims, eight unrelated victims, a family of two children, or a family of eight children. We compared mean donations and self-reported affect between conditions using ANOVAs and planned contrasts. We expected that the decline in response observed in our previous studies would be reversed or mitigated by the entitativity manipulations. More specifically, we expected that mean donations and positive affect would be higher in conditions where the children were portrayed as (related) group (high entitativity) compared to when the same children were described as unrelated individuals.

#### Method

Students in a Swedish university (*N* = 131, 82 females, mean age 24.5, *SD* 5.1) participated in the study. A between-subjects design was used where participants were randomly allocated to one of five conditions: 1) a single child, 2) two unrelated children, 3) eight unrelated children, 4) two related children, and 5) eight related children. In the single-child condition, participants were given a photograph and a name and instructed that: “you can help [name] with your donation.” In the two-unrelated-children condition, participants were also given photographs and names, but each child was depicted in a separate picture. Participants were instructed that: “you can help [name] and [name] with your donation.” A similar procedure was used for the eight-unrelated-children condition. In the related-children conditions, participants were shown the same children, all together in the same photograph, and were instructed that “you can help the family consisting of [name] and [name] and… with your donation.” Participants were asked to indicate how much they would be willing to contribute to the cause (a hypothetical donation) followed (on a separate page) by ratings of perceived need “How much does/do the child/ren need your help?” rated on a 1 (*not at all*) to 5 (*very much*) scale, as well as by ratings of positive and negative affect (“How positive/negative do you feel about helping the child/ren?” on a 0 (*not at all*) to 5 (*very much*) scale).

#### Results and discussion

Consistent with our previous results, donations decreased linearly with an increasing number of unrelated victims (Table 1; linear trend *F*(4,126) = 4.25, *p*<.01). Similarly, positive affect decreased with an increasing number of unrelated victims (*F*(4, 126) = 2.22, *p*<.05). More importantly, both the critical contrasts between two unrelated/two related children (*t*(54) = 1.88, *p*<.05) and eight unrelated/eight related children (*t*(51) = 2.6, *p*<.05) were significant for donations. As can be seen in Table 1, donations increased when the same number of children were described as a coherent unit–a family. A main effect of related versus unrelated condition on need ratings was observed (*F*(4, 130) = 2.93, *p*<.05), but only the eight-children contrast was significant (*t*(51) = 2.3, *p*<.05) with higher need ratings for the related condition. Similarly, both positive (*t*(49) = 2.1, *p*<.05) and negative affect (*t*(49) = 3.3, *p*<.01) were higher in the eight-related-children condition than in the eight-unrelated-children condition. No other comparisons were significant.

**Table 1:** Mean WTC, Need, and Positive Affect (PA) and Negative Affect (NA) (Study 3).

	Unrelated			Related	
Dependent	One child	Two children	Eight children	Two children	Eight children
WTC	31.8	25.9	20.0	32.9	32.1
Need	3.0	2.7	2.9	3.1	3.8
PA	3.4	3.0	2.6	3.1	3.3
NA	2.0	2.3	2.4	2.7	3.2

Study 3 thus provided further support for the notion that a single individual is processed differently than a group of individuals. First, we replicated the finding that, as the number of children increases, donations and positive affect decrease. Second, by using entitativity manipulations whereby several children were perceptually (appearing in the same photograph) and psychologically (described as “a family”) grouped into a single unit, we were able to show that donations and affect were comparable to that of a single individual. These findings support the notion that compassion fade is an affective phenomenon where feelings are greatest for single individuals, or groups perceived as individual units. These findings not only extend our understanding of the psychology underlying compassion fade, but also suggest ways to combat loss of feeling as need increases. Thus far, we have examined compassion fade in a positive frame–life saving. In Study 4, we will examine whether compassion fade extends to frames involving losses of life.

### Study 4: Singularity Effect and Compassion Fade in Loss Frames

The studies here, and in previous research, have shown that people will go to great lengths to save a single identified victim [1], [29]. Much of the current research on identifiable victim effects or singularity has been examined in the context of positive frames, via providing aid through donations or charitable behavior. In Study 4 we consider compassion fade in loss frames, in the context of mitigating possible deaths. We hypothesize that it should also be the case that people will be most averse to the death of a single identified victim. Study 4 used hypothetical crisis scenarios to test the sensitivity to potential losses of life.

#### Method

Students at the University of Oregon (*N* = 559) completed a short survey for class credit. Although demographic information was not collected, similar university samples have consisted of approximately 60% females with a mean age of 19.5 (*SD* 2.2). Participants were asked to consider the following scenario:

Imagine you are a member of a civil defense committee that is considering contingency plans in the event of various emergencies. One emergency under discussion is the following:

A train carrying a very toxic chemical derails and the storage tanks begin to leak. The threat of explosion and lethal discharge of poisonous gas is imminent.

Two possible actions are being considered. Read them and indicate your preference and opinion of each.

Option A: would contain the threat but carries a.5 probability of losing 60 lives and a.5 probability of losing 40 lives

Option B: would contain the threat but carries a.5 probability of losing 150 lives and a.5 probability of losing 0 lives [This option varied by condition.]

Which option do you prefer?

We used a between-subjects design in which subjects were assigned one of the following four choices for option B (option A remained the same for all conditions and always offered a smaller expected loss of life than Option B).

Condition 1).5 probability of losing 150 lives and a.5 probability of losing 0 lives.

Condition 2).5 probability of losing 150 lives and a.5 probability of losing 1 life (John Davis, a chemical engineer that would play a crucial but dangerous role in the cleanup).

Condition 3).5 probability of losing 150 lives and a.5 probability of losing 2 lives (John Davis and Richard Carey, chemical engineers that would play a crucial but dangerous role in the cleanup).

Condition 4).5 probability of losing 150 lives and a.5 probability of losing 3 lives (John Davis, Richard Carey, and Josh Burns, chemical engineers that would play a crucial but dangerous role in the cleanup).

Fictitious names and stock portraits were used to identify the possible victims in option B. Participants chose either option A or option B, rated their strength of preference for their chosen option, and gave a brief explanation for their choice. Strength of preference was given on a five-point scale, ranging from *No preference* to *Very strongly prefer*. Option A remained the same for all conditions and always offered a smaller expected loss of life than Option B. After indicating their choice, participants gave a brief reason for it.

#### Results and discussion

**Figure 3:**
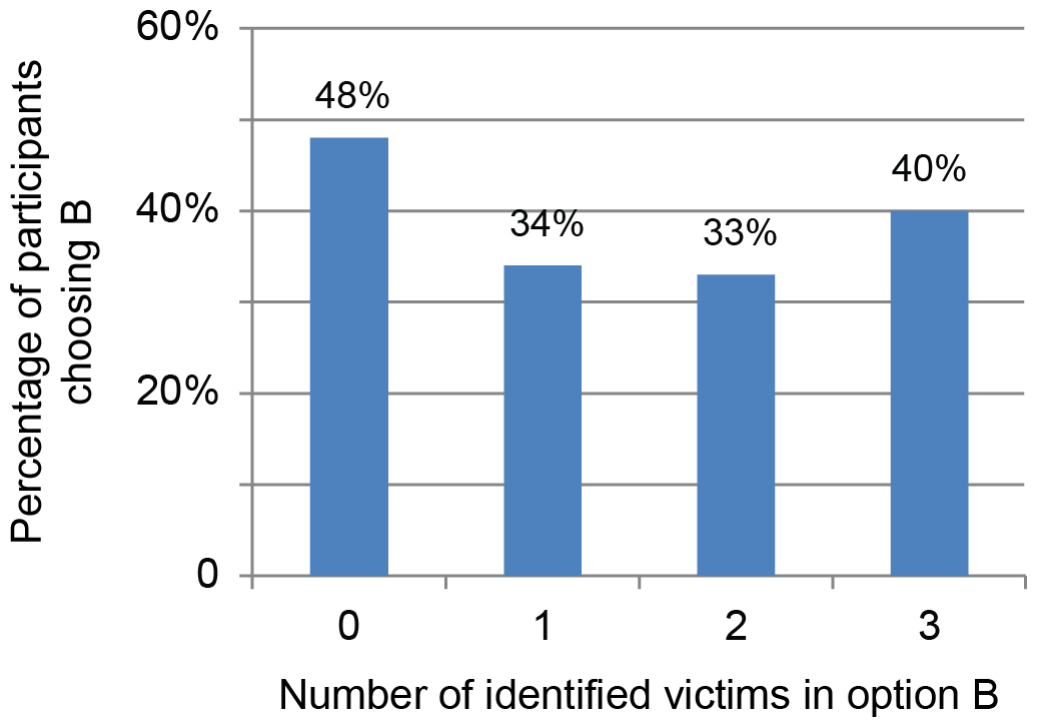
Preferences shifted when identified victims were presented in loss-of-life gambles.

Figure 3 shows the percentage of participants choosing Option A or Option B for each condition. An omnibus effect of condition was found for the percentage of choices (*X* ^2^ (1, *N* = 559) = 8.03, *p*<.05). The percentage of people choosing A or B was close to 50% in the zero-identified-victim Condition 1. Based on the self-reported explanations of choice, many respondents were enticed by the possibility of zero deaths, despite the high risk of 150 deaths. Consistent with the singularity effect, adding one identified victim to option B created a strong shift in choices and preferences. Option B was chosen significantly less often (*X* ^2^ (1, *N* = 280) = 5.33, *p*<.05) and was considered significantly less preferable (*t*(113) = 4.39, *p*<.001) in the single-victim condition, compared to the zero-loss-of-life condition, despite the fact that the difference in negative expected value was negligible between the two options (–75.5 lives compared to –75).

Increasing the number of identified victims from 1 to 2 slightly decreased the percentage of respondents who chose option B (34% to 33%), but significantly increased strength of preference ratings for option B (*t*(92) = –2.35, *p*<.05). Increasing the number of identified victims from two to three *increased* the proportion of times Option B was chosen (33% to 40%) and further increased strength of preference ratings for option B, although these differences did not reach statistical significance.

Preferences for options A and B were then recoded into a single continuous scale (Option A: *No preference* = 0, *Slightly prefer* = –1, *Prefer* = –2, *Strongly prefer* = –3, *Very strongly prefer* = –4; Option B: *No preference* = 0, *Slightly prefer* = 1, *Prefer* = 2, *Strongly prefer* = 3, *Very strongly prefer* = 4). A main effect of condition was found (*F*(3,546) = 4.39, *p*<.01) as well as negative quadratic trend in preference across conditions (*F*(1,546) = 6.41, *p*<.05). The negative quadratic trend complements the choice data, indicating a large drop in preference for option B from zero identified victims to one, but flattening (and slightly increasing) as the number of identified victims increases.

These results mirror the donation findings, but in the context of loss frames. Participants were much less likely to risk the death of a single identified victim than when no identified victim was present, even though the alternative posed the loss of at least 40 lives! Participants were slightly more likely to risk the death of three identified victims than one identified victim. The results are consistent with both a psychophysical function for loss frames and a possible decline or fading of compassion as the number of identified victims at risk increased. Perhaps if the number of identified victims continued to increase, the percentage of people choosing to risk the identified victims’ lives might also increase and eventually be identical to the zero-victim condition.

## General Discussion and Conclusions

The results from four studies show that affective feelings about charitable causes were strongest for a single endangered person and began to decline as the number in danger grew larger. In support of compassion fade, both self-report and physiological measures of affect showed that positive affect declined substantially when the group size was two or more. This decrease in positive affect was related to lower donations. A decrease in positive affect with increasing numbers at risk is consistent with research in social psychology showing differential processing of individuals and groups [36], research on decline in object attachment when the number of objects increases [37], [45], and research on decline of attention and sympathy as more people enter the visual field [14].

**Figure 4:**
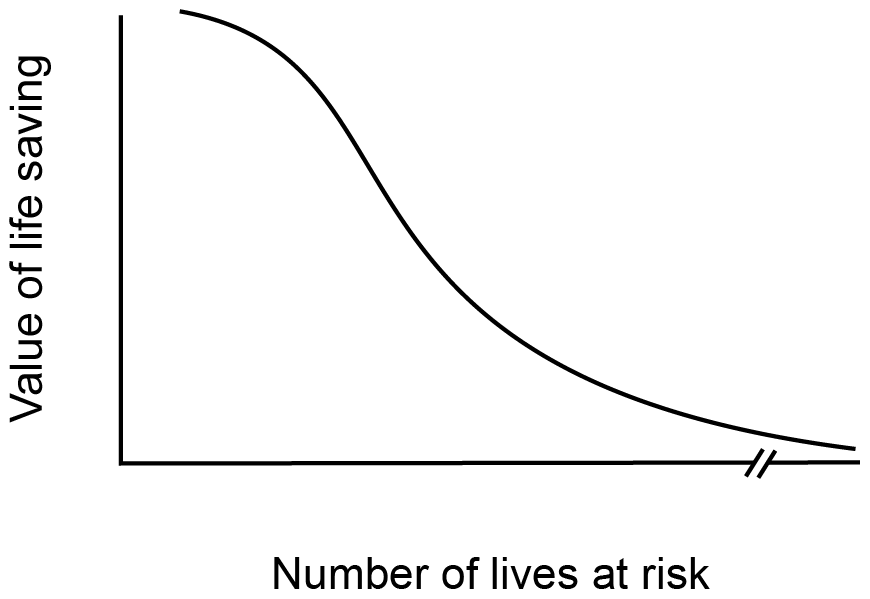
A model depicting psychic numbing–compassion fade–when valuing the saving of lives.

Previous studies [30] have demonstrated the singularity effect showing that a single individual in need (compared to a larger group) elicits more compassion and donations. Our first three studies replicated and extended these findings by showing that the singularity effect already begins to break down as we move from one to two persons in need. This finding hints at a disturbing psychological tendency. Our capacity to feel positive affect for people in need may be limited. When lives are at stake, feelings necessary to motivate lifesaving action may peak at *N* = 1 person. Attention, feelings, and response may begin to decline or fade at *N* = 2, eventually collapsing at some higher value of *N* that is perceived as merely “a statistic.” (See Figure 4).

There are several ways to account for compassion fade. Our results suggest that people begin to lose affective attachment as the number in need increases. We argue that it may be natural and relatively easy to empathize and feel compassion with a single identified individual, but that it is difficult to “scale up” this emotion when we need to consider more than one individual. In fact, as the number in need increases, we may find it more difficult to empathize, but at the same time feel more negative emotion. Cameron and Payne [51] showed that as the number of lives in need of help increases, people experience negative affect and attempt to regulate these negative feelings by turning their attention away from the problem. A similar prediction can be derived from Batson’s empathic concern/personal distress framework – as the number of individuals in need increase the more personal distress is experienced [38]. Stronger egocentric motivations (i.e. desire to feel better) with increased personal distress may lead to motivated affect regulation and decreased giving [52].

The findings in this paper are partly compatible with these explanations for compassion fade. More specifically, we found in both in Studies 1 and 2 that physiological and self-reported measures of negative affect did not decrease but instead tended to increase as the number of children in need increased. However, negative affect was not predictive of donation behavior in either study. Instead, and counter to what may be expected from motivated down-regulation of negative affect, we found that the single individual elicits *positive affect* that decreases as the number of children in need increase. This decrease in positive affect was predictive of reduced donations. Motivated emotion regulation cannot easily account for this finding. Instead, our data suggest that losses of attention and positive emotion are critical mechanisms underlying compassion fade. This finding also runs counter to a possibility raised by the empathic concern/personal distress framework - that a single identified victim elicits the highest distress and therefore should be associated with the lowest levels of giving [38]. Consistent with our psychophysiological findings implicating loss of positive affect, recent brain-imaging research has shown that identifiability effects (giving more to single identified compared with unidentified victims) are uniquely predicted by changes in reward circuitry in the nucleus accumbens [13]). Our findings suggest that valanced affect is a driver of compassion fade. It is however possible that different distinct emotional states associated with different approach-avoidance motivations may override, attenuate, or amplify compassion fade [43], [44]. Future research should address this issue more closely.

The natural and easy way to deal with moral issues is to rely on our intuitions. “How important is this person’s or this group’s need for assistance? Well, how important does it feel?” We can also apply reasoned deliberation to guide us but, as Haidt [53], Greene [54], Sunstein [54] has demonstrated (see [55] for alternative conceptualizations), moral intuition usually comes first and dominates moral judgment unless we make an effort to critique and, if necessary, override our intuitive affective feelings. Left to its own devices, moral intuition will likely favor individual victims and under-react to large-scale crises [1]. Our sizable capacity to care for others may not be engaged unless we find ways to overcome compassion fade. In Study 3, we showed that describing several children as a single unit raised the level of donations and affect to the level of the single individual (see also [50]). This approach may therefore be fruitful in raising the level of help given to the many in need.

Unfortunately, we are not always in a position of saving lives. In many real-world situations, decisions must be made to mitigate death [1]. For example, war strategists must make tactical decisions in the face of collateral deaths of many innocent lives; emergency response teams frequently encounter tragic situations in which not all victims can be saved [2]. We must consider that decision makers may be vulnerable to the same biases of singularity in loss-of-life scenarios as found in life-saving scenarios. Study 4 showed that, when choosing hypothetical contingency plans to mitigate deaths, people are averse to an option that risks the death of a single identified victim. When participants were asked to explain their choice, one subject stated, “One specific death made it seem all too real.” Similar to our donation study findings, the loss of an identified victim creates an affect-rich scenario. But those feelings are spread more and more thinly as the number of victims increases.

There are, of course, examples of sizable contributions of aid to the thousands of victims of a natural disaster that seem to run counter to compassion fade. Examination of these events show they differ in a number of ways from the life-saving cases studied here. First, they represent acute events, with a relatively clear time course, after which much of the aid is given to enable recovery. Second, these acute catastrophes are accompanied by massive, comprehensive, and vivid media coverage, including dramatic personal stories of identified victims [2]. Donors can understand the distress experienced by the victims and empathize with them [3]. This differs greatly from the scenarios studied here, involving typically invisible crises of individuals afflicted with chronic conditions of poverty such as hunger, malnutrition, and disease. Natural disasters play out over a long enough period of time for slow thinking to comprehend the seriousness of the suffering and devastation they cause [3]. Our donors, in contrast, are given only a brief moment to make their decisions. Finally, as large as the donations seem to be (e.g., more than $4 billion after Hurricane Katrina), they pale in comparison to the economic costs (more than $250 billion for Katrina), not to mention the human costs. As the ongoing emergency fades, efforts to prevent or mitigate future disasters meet the same psychic numbness we document here.

### Implications for Theory and Applications

**Figure 5:**
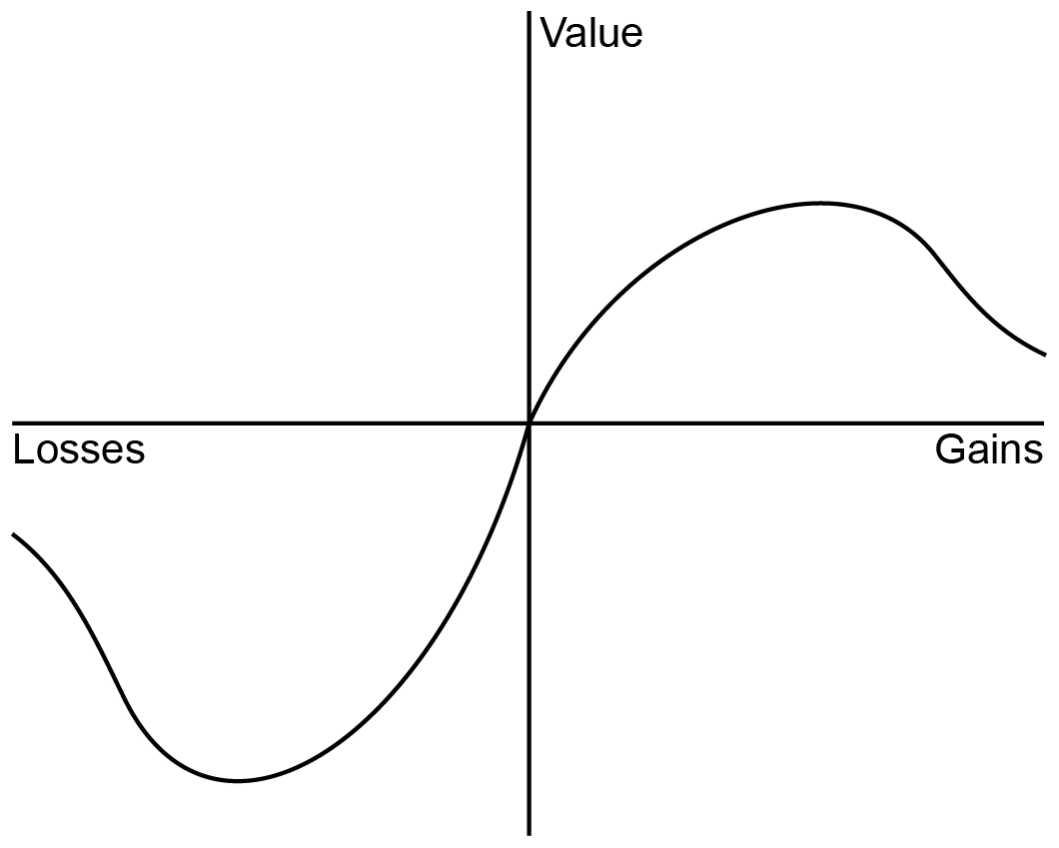
A modified value function.

At a theoretical level, these results suggest that important descriptive decision-making accounts such as prospect theory may not always adequately describe how people value human lives [4], [5]. We propose that, for life-saving decisions, both the gain (Studies 1–3) and loss domain (Study 4) of the value function may not only be characterized by a decreased sensitivity as magnitude increases (the psychophysical function in Figure 1), but may sometimes even show a decline in value (Figure 5). Figure 5 hypothesizes a decreased sensitivity and at some point decreasing value in both the loss and gain domains (an inverted U-shape in the gain domain; U-shaped for losses) reflecting eventual loss of value (following initial decrease in sensitivity).

There is considerable evidence outside the field of judgment and decision making for a value function following such an inverted U-shape function in the gain domain. While it is difficult to find evidence of the negative utility of increases in monetary wealth, other forms of basic economic behavior can follow a declining function even to the point of collapse. For example, food consumption often follows this trajectory where the value of initial food intake is very high. After attaining some level of satiation (a psychophysical function consistent with prospect theory), further food intake may no longer be attractive (i.e. the value is changing). Importantly, at some point (a threshold that may vary with individuals and over time and contexts) *the value* of further intake is going to start to decline (i.e. a negative value), perhaps precipitously [55], [58]. We believe that such a model can describe how we value magnitudes more generally in at least some domains, including valuation of lives. While the diminishing sensitivity to magnitude in prospect theory may be explained (at least in part) by diminishing sensitivity to abstract numbers ([59], the further collapse in domains such as valuation of lives may be determined by decreased positive affect to those additional lives.

How can this tendency to underreact be overcome? Recent research suggests that the singularity effect may be used to boost giving. Hsee et al. [60] found that merely asking donors to indicate a hypothetical amount for helping one needy person, before asking donors to decide how much to donate for all of those in need, increased donations for the group.

Ultimately, thoughtful deliberation, what Kahneman [6] calls slow thinking, may be necessary to alert us to an undesired disconnect between the high value we place on individual lives and our neglect of populations at risk [30], [61], [62]. Perhaps this deliberative perspective will impress upon us the need to create institutional mechanisms that doggedly pursue the hard measures needed to combat mass tragedies when our attention strays and our numbed feelings lull us into complacency [3], [63].
